# Supplementary material for: Novel Glycosylation by Amylosucrase to Produce Glycoside Anomers
Source: Biology (Basel). 2022 May 27;11(6):822. doi: 10.3390/biology11060822 (PMC9220500; doi:10.3390/biology11060822)
Supplement: Supplementary file 1 [file biology-11-00822-s001.zip › biology-1739449-supplementary.pdf]

Contents

Table S1. ^{1}H and ^{13}C NMR assignments in pyridine-d_5 at 700 and 175 MHz for compounds (**1**; *α*-glucosyl-(2→26)-GAF and *β*-glucosyl-(2→26)-GAF). (*δ* in ppm, *J* in Hz).

Table S2. ^{1}H and ^{13}C NMR assignments in pyridine-d_5 at 700 and 175 MHz for compounds (**2**; *α*-glucosyl-(2→26)-GAA and *β*-glucosyl-(2→26)-GAA). (*δ* in ppm, *J* in Hz).

Table S3. ^{1}H and ^{13}C NMR assignments in pyridine-d_5 at 700 and 175 MHz for compounds (**3**; *α*-glucosyl-(2→26)-GAG and *β*-glucosyl-(2→26)-GAG). (*δ* in ppm, *J* in Hz).

Figure S1. High-performance liquid chromatography (HPLC) results of the biotransformation products of ganoderic acid F (GAF) using amylosucrase from *Deinococcus geothermalis* (*Dg*AS) enzyme at different pH levels.

Figure S2. The mass-mass analysis of compound (**1**) at the negative mode.

Figure S3. 1D NMR spectrum (^{1}H-NMR, 700 MHz, pyridine-d_5) of the compound (**1**).

Figure S4. 1D NMR spectrum (^{13}C-NMR, 175 MHz, pyridine-d_5) of the compound (**1**).

Figure S5. 1D NMR spectrum (DEPT-135, 175 MHz, pyridine-d_5) of the compound (**1**).

Figure S6. 2D NMR spectrum (^{1}H-^{13}C HSQC, 700 MHz, pyridine-d_5) of the compound (**1**).

Figure S7. 2D NMR spectrum (^{1}H-^{13}C HMBC, 700 MHz, pyridine-d_5) of the compound (**1**).

Figure S8. 2D NMR spectrum (^{1}H-^{1}H COSY, 700 MHz, pyridine-d_5) of the compound (**1**).

Figure S9. 2D NMR spectrum (^{1}H-^{1}H NOESY, 700 MHz, pyridine-d_5) of the compound (**1**).

Figure S10. HPLC results of the biotransformation products of GAA (a) and GAG) (b) using *Dg*AS enzyme at different pH levels.

Figure S11. The mass-mass analysis of compound (**2**) at the negative mode.

Figure S12. The mass-mass analysis of compound (**3**) at the negative mode.

Figure S13. 1D NMR spectrum (^{1}H-NMR, 700 MHz, pyridine-d_5) of the compound (**2**).

Figure S14. 1D NMR spectrum (^{13}C-NMR, 175 MHz, pyridine-d_5) of the compound (**2**).

Figure S15. 1D NMR spectrum (DEPT-135, 175 MHz, pyridine-d_5) of the compound (**2**).

Figure S16. 2D NMR spectrum (^{1}H-^{13}C HSQC, 700 MHz, pyridine-d_5) of the compound (**2**).

Figure S17. 2D NMR spectrum (^{1}H-^{13}C HMBC, 700 MHz, pyridine-d_5) of the compound (**2**).

Figure S18. 2D NMR spectrum (^{1}H-^{1}H COSY, 700 MHz, pyridine-d_5) of the compound (**2**).

Figure S19. 2D NMR spectrum (^{1}H-^{1}H NOESY, 700 MHz, pyridine-d_5) of the compound (**2**).

Figure S20. 1D NMR spectrum (^{1}H-NMR, 700 MHz, pyridine-d_5) of the compound (**3**).

Figure S21. 1D NMR spectrum (^{13}C-NMR, 175 MHz, pyridine-d_5) of the compound (**3**).

Figure S22. 1D NMR spectrum (DEPT-135, 175 MHz, pyridine-d_5) of the compound (**3**).

Figure S23. 2D NMR spectrum (^{1}H-^{13}C HSQC, 700 MHz, pyridine-d_5) of the compound (**3**).

Figure S24. 2D NMR spectrum (^{1}H-^{13}C HMBC, 700 MHz, pyridine-d_5) of the compound (**3**).

Figure S25. 2D NMR spectrum (^{1}H-^{1}H COSY, 700 MHz, pyridine-d_5) of the compound (**3**).

Figure S26. 2D NMR spectrum (^{1}H-^{1}H NOESY, 700 MHz, pyridine-d_5) of the compound (**3**).

Table S1. ^{1}H and ^{13}C NMR assignments in pyridine-d_5 at 700 and 175 MHz for compounds (**1**; *α*-glucosyl-(2→26)-GAF and *β*-glucosyl-(2→26)-GAF**)**. (*δ* in ppm, *J* in Hz).

position	compound **(1)**	position	compound **(1)**
1	37.7	1	37.7
2	34.2	2	34.2
3	214.6	3	214.6
4	46.9	4	46.9
5	50.7	5	50.7
6	34.0	6	34.0
7	199.0	7	199.0
8	150.2	8	150.2
9	146.6	9	146.6
10	39.5	10	39.5
11	194.9	11	194.9
12	79.5	12	79.5
13	48.0	13	48.0
14	59.1	14	59.1
15	206.1	15	206.1
16	38.2	16	38.2
17	44.9	17	44.9
18	18.8	18	18.8
19	12.1	19	12.1
20	30.0	20	29.9
21	21.7	21	21.7
22	48.7	22	48.8
23	208.2	23	207.9
24	46.8	24	46.8
25	35.5	25	35.5
26	176.2	26	175.4
27	17.2	27	17.2
28	20.2	28	20.2
29	27.2	29	27.2
30	20.2	30	20.2
O$\underline{C}$OCH_3	170.1	O$\underline{C}$OCH_3	170.1
OCO$\underline{C}H_3$	21.7	OCO$\underline{C}H_3$	21.7
α-glucose		*β*-glucose	
1'	91.2 (6.05, d, *J*=3.5 Hz)	1'	96.5 (5.38, d, *J*=7.7 Hz)
2'	75.9 (5.50, dd, *J*=3.5, 9.8 Hz)	2'	77.3 (5.65, dd, *J*=7.7, 9.1 Hz)
3'	72.3 (4.94, m)	3'	76.5 (4.27, m)
4'	72.8 (4.32, m)	4'	73.7 (4.63, m)
5'	73.4 (4.81, m)	5'	71.0 (4.33, m)
6'	62.9 (4.41, m; 4.53, m)	6'	62.7 (4.36, m; 4.56, m)

Table S2. ^1H and ^{13}C NMR assignments in pyridine-d_5 at 700 and 175 MHz for compounds (**2**; *α*-glucosyl-(2→26)-GAA and *β*-glucosyl-(2→26)-GAA). (*δ* in ppm, *J* in Hz).

position	compound **(2)**	position	compound **(2)**
1	36.0	1	36.0
2	34.6	2	34.6
3	216.2	3	216.2
4	47.0	4	47.0
5	48.9	5	48.9
6	29.6	6	29.6
7	68.7	7	68.7
8	161.5	8	161.5
9	139.9	9	139.9
10	38.3	10	38.3
11	200.0	11	200.0
12	52.4	12	52.4
13	46.7	13	46.7
14	54.7	14	54.7
15	72.2	15	72.2
16	36.9	16	36.9
17	49.8	17	49.8
18	19.7	18	19.7
19	17.5	19	17.5
20	33.0	20	32.9
21	20.3	21	20.3
22	49.8	22	50.0
23	208.7	23	208.3
24	47.0	24	46.9
25	35.4	25	35.5
26	176.2	26	175.5
27	17.2	27	17.3
28	19.6	28	19.6
29	27.2	29	27.2
30	20.8	30	20.8
α-glucose		*β*-glucose	
1'	91.1 (6.04, d, *J*=3.5 Hz)	1'	96.5 (5.37, d, *J*=7.7 Hz)
2'	75.9 (5.48, dd, *J*=3.5, 9.8 Hz)	2'	77.2 (5.64, t, *J*=8.4 Hz)
3'	72.2 (4.94, t, *J*=9.1 Hz)	3'	76.4 (4.28, t, *J*=9.1 Hz)
4'	72.8 (4.30, t, *J*=9.1 Hz)	4'	75.2 (4.62, t, *J*=9.1 Hz)
5'	73.7 (5.80, m)	5'	71.1 (4.34, m)
6'	62.9 (4.42, m; 4.52, dd, *J*=2.8, 11.9 Hz)	6'	62.7 (4.56, m; 4.56, dd, *J*=2.1, 11.9 Hz)

Table S3. ^{1}H and ^{13}C NMR assignments in pyridine-*d*5 at 700 and 175 MHz for compounds (**3**; *α*-glucosyl-(2→26)-GAG and *β*-glucosyl-(2→26)-GAG**)**. (*δ* in ppm, *J* in Hz).

position	compound **(3)**	position	compound **(3)**
1	35.0	1	35.0
2	28.1	2	28.1
3	77.5	3	77.5
4	39.2	4	39.2
5	49.7	5	49.7
6	28.4	6	28.4
7	66.3	7	66.3
8	158.8	8	158.8
9	142.5	9	142.5
10	38.8	10	38.8
11	200.8	11	200.8
12	79.2	12	79.2
13	52.1	13	52.1
14	60.0	14	60.0
15	216.6	15	216.6
16	38.8	16	38.8
17	46.6	17	46.6
18	19.0	18	19.0
19	12.8	19	12.8
20	28.8	20	28.9
21	22.0	21	21.9
22	48.8	22	48.9
23	208.6	23	208.3
24	46.7	24	46.7
25	35.4	25	35.5
26	176.3	26	176.5
27	17.2	27	17.2
28	23.6	28	23.6
29	28.7	29	28.7
30	16.3	30	16.3
α-glucose		*β*-glucose	
1'	91.1 (6.05, d, *J*=2.8 Hz)	1'	96.5 (5.38, d, *J*=8.4 Hz)
2'	75.9 (5.47, dd, *J*=3.5, 9.8 Hz)	2'	77.2 (5.64, t, *J*=8.4 Hz)
3'	72.2 (4.95, t, *J*=9.8 Hz)	3'	76.5 (4.21, t, *J*=9.1 Hz)
4'	72.7 (4.30, t, *J*=9.1 Hz)	4'	75.2 (4.62, t, *J*=9.1 Hz)
5'	73.7 (4.81, m)	5'	71.1 (4.34, m)
6'	62.9 (4.40, m; 4.56, dd, *J*=2.1, 11.9 Hz)	6'	62.7 (4.35, m; 4.52, dd, *J*=2.1, 11.9 Hz)

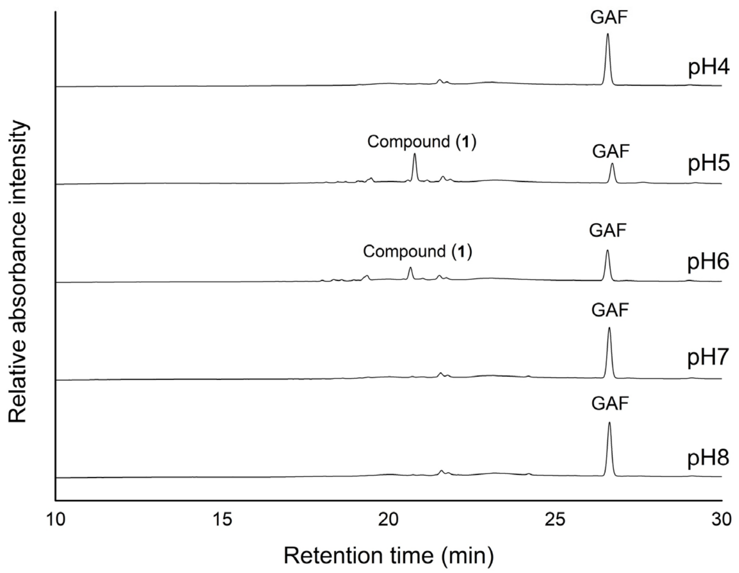

Figure S1. High-performance liquid chromatography (HPLC) results of the biotransformation products of ganoderic acid F (GAF) using amylosucrase from *Deinococcus geothermalis* (*Dg*AS) enzyme at different pH levels. The biotransformation condition was 1 mg/mL of GAF, 25 g/mL of *Dg*AS, and 1500 mM of sucrose at 50 mM of acetate buffer (pH 4 and 5), phosphate buffer (pH 6 and 7), or Tris buffer (pH 8) and 40 °C for 24 h. After incubation, the biotransformation products were analyzed using HPLC. The detailed reaction conditions and the HPLC procedure are described in the Materials and Methods section.

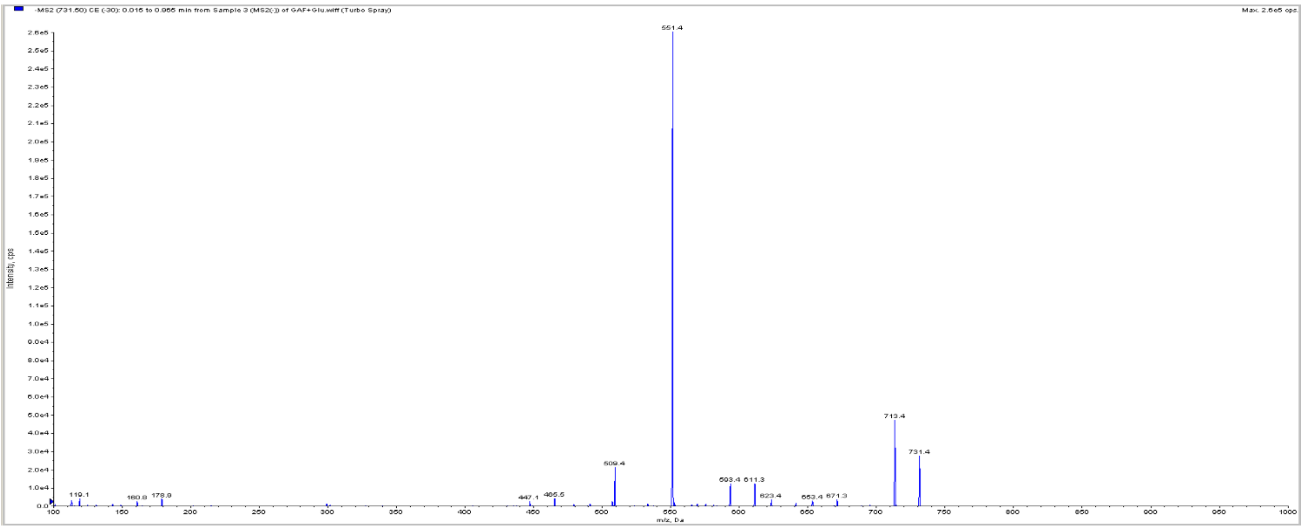

Figure S2. The mass-mass analysis of compound (**1**) at the negative mode. A significant signal at m/z 731.4 showed the corresponding m/z signal of molecular weight 732 of GAF-glucoside (570+180-18) at the negative mode.

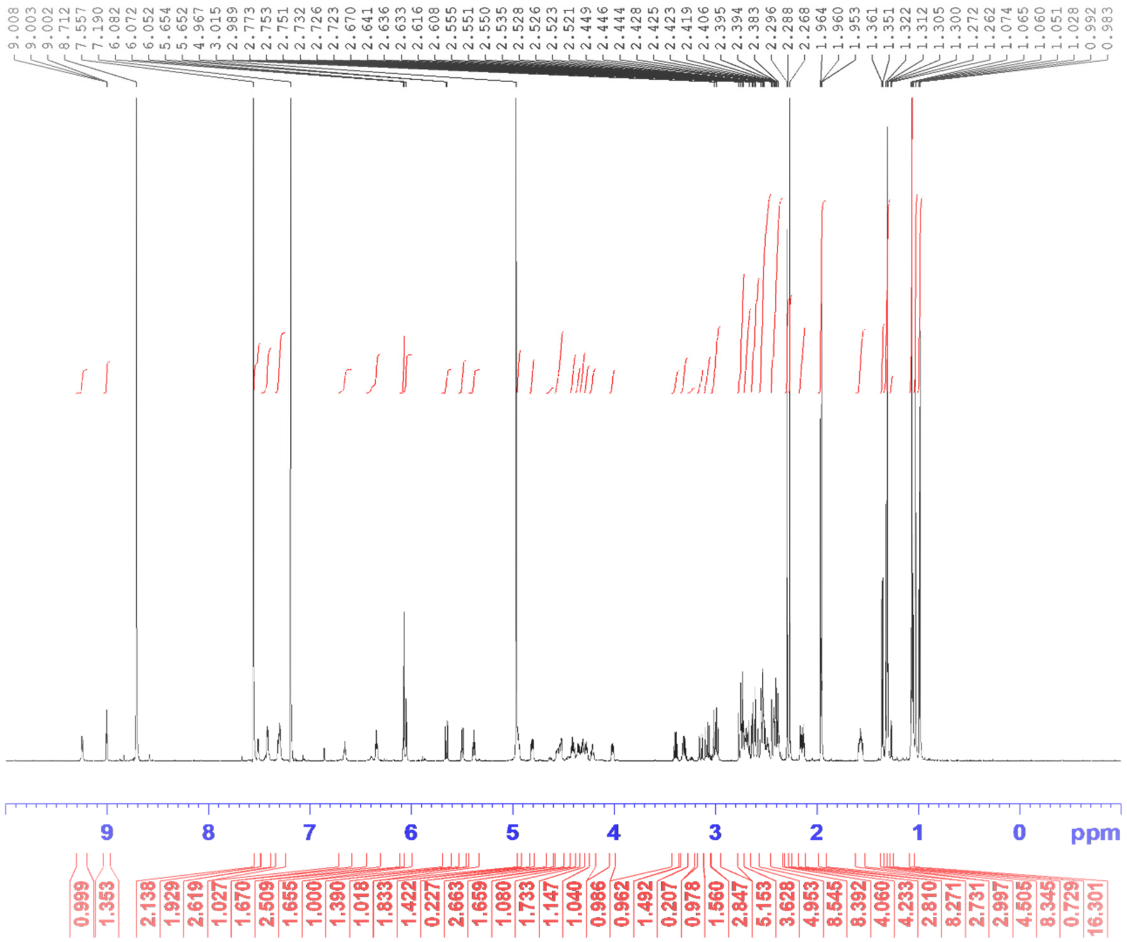

Figure S3. 1D NMR spectrum (^{1}H-NMR, 700 MHz, pyridine-d_5) of the compound (**1**).

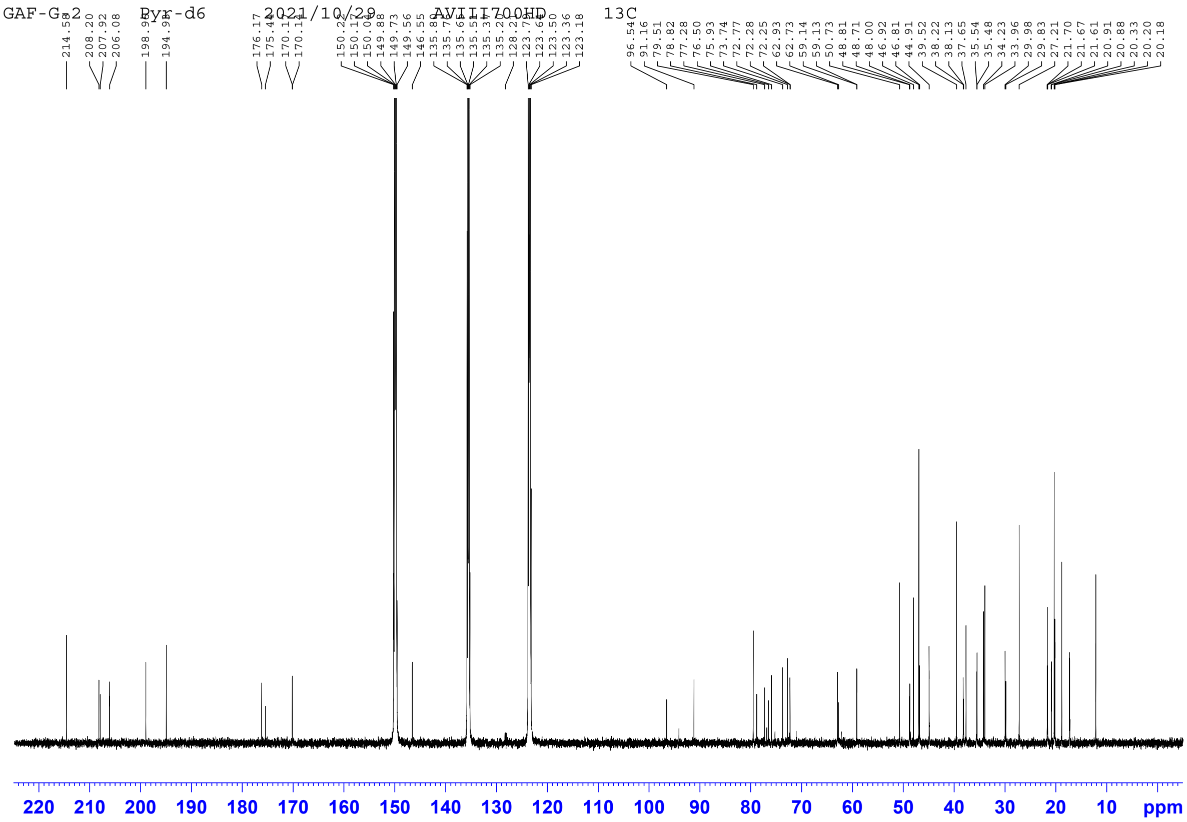

Figure S4. 1D NMR spectrum (^{13}C-NMR, 175 MHz, pyridine-d_5) of the compound (**1**).

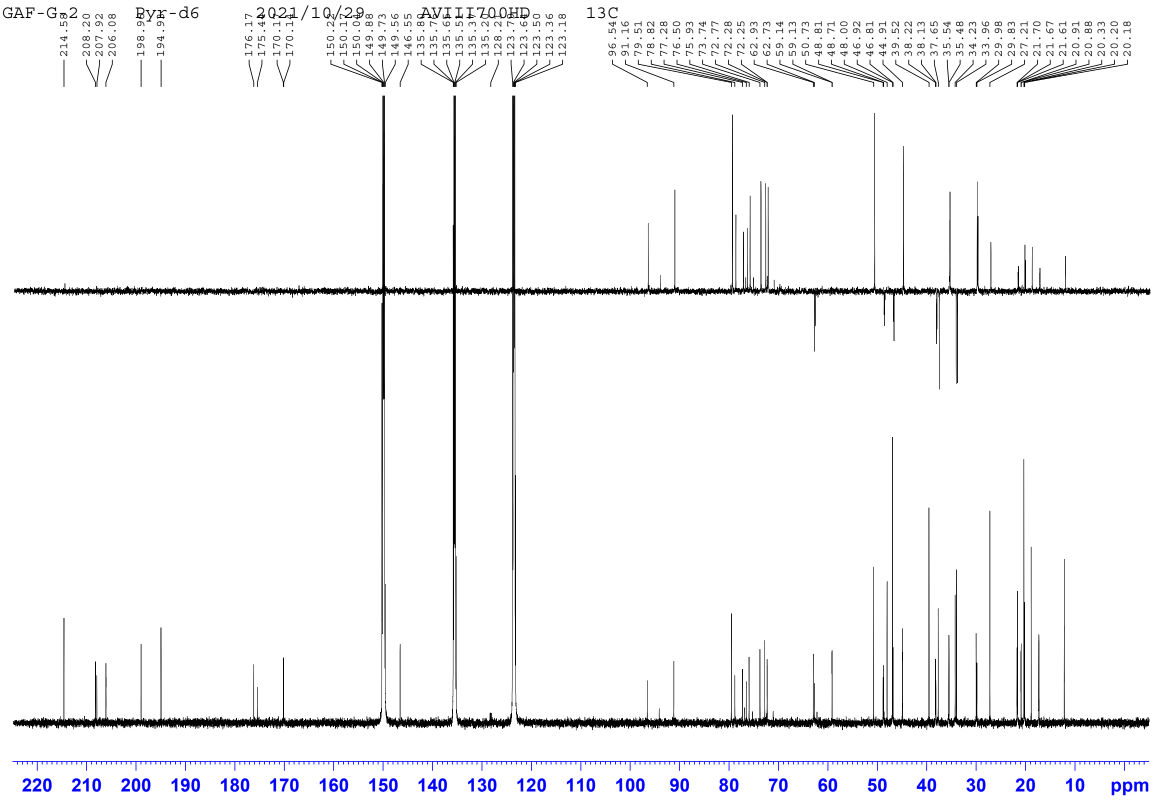

Figure S5. 1D NMR spectrum (DEPT-135, 175 MHz, pyridine-d_5) of the compound (**1**).

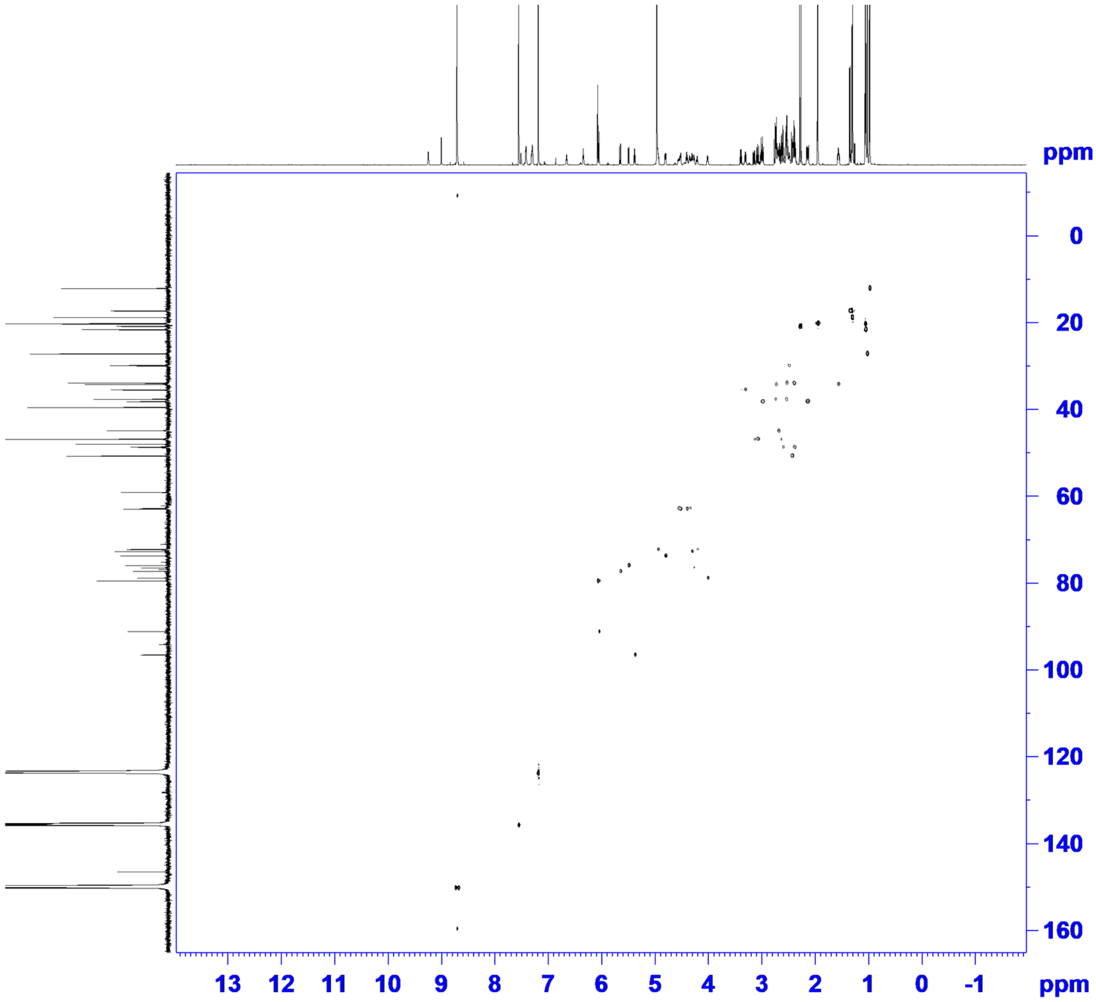

Figure S6. 2D NMR spectrum (^1H-^{13}C HSQC, 700 MHz, pyridine-d_5) of the compound (**1**).

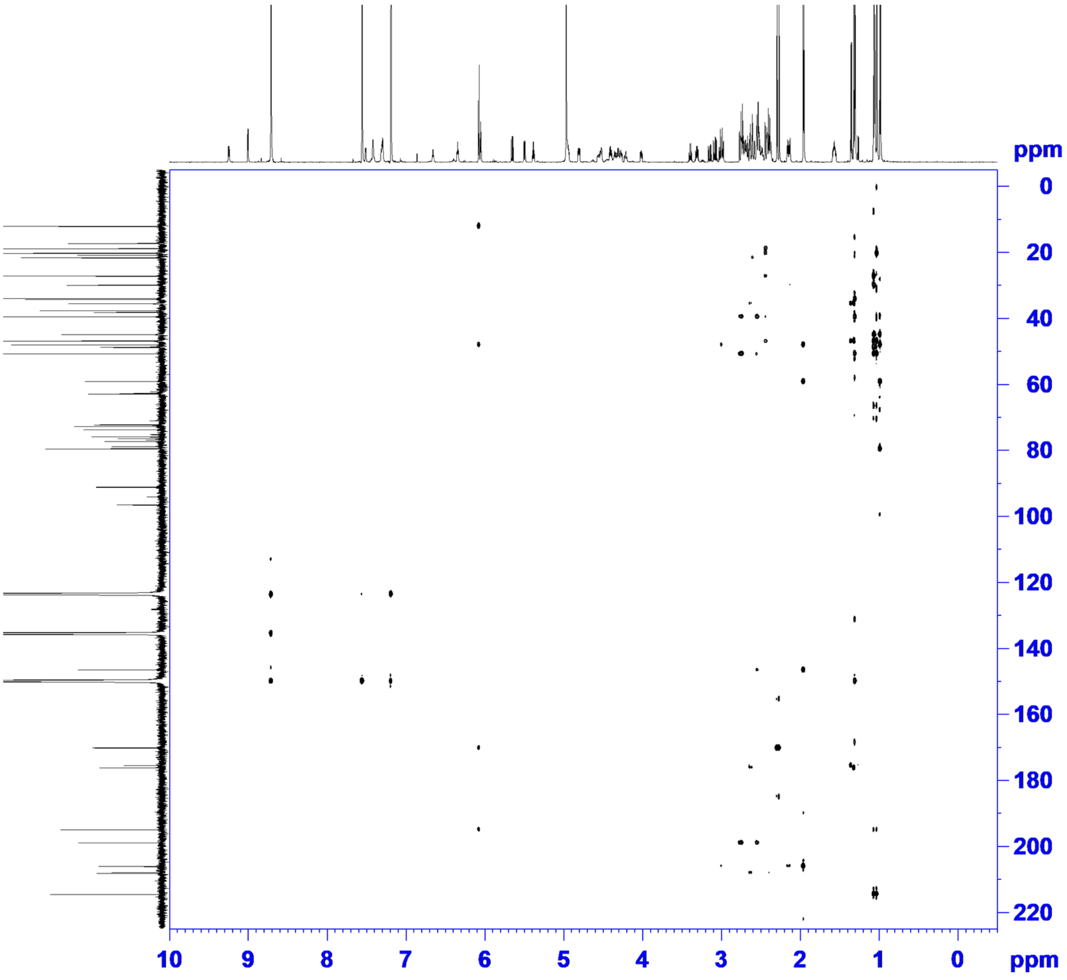

Figure S7. 2D NMR spectrum (^{1}H-^{13}C HMBC, 700 MHz, pyridine-d_5) of the compound (**1**).

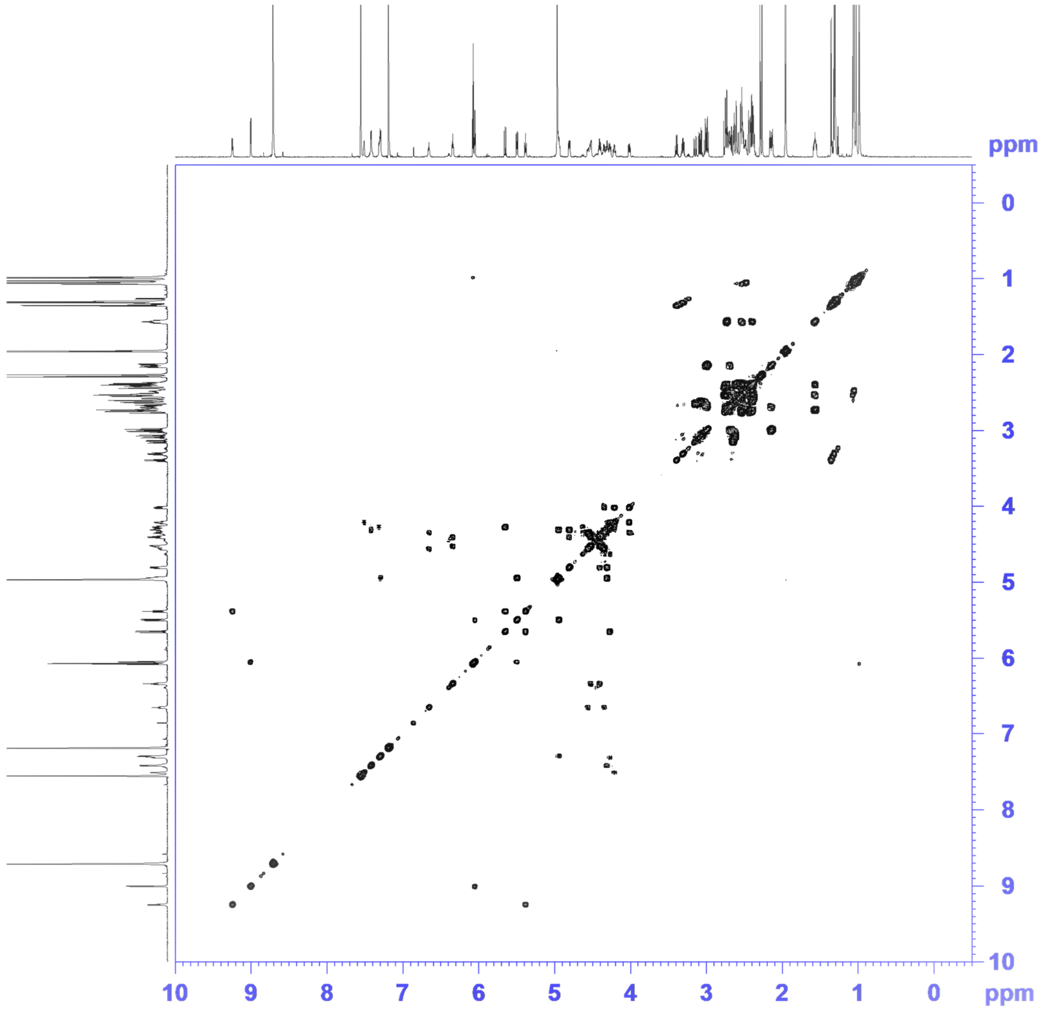

Figure S8. 2D NMR spectrum (^1H-^1H COSY, 700 MHz, pyridine-d_5) of the compound (**1**).

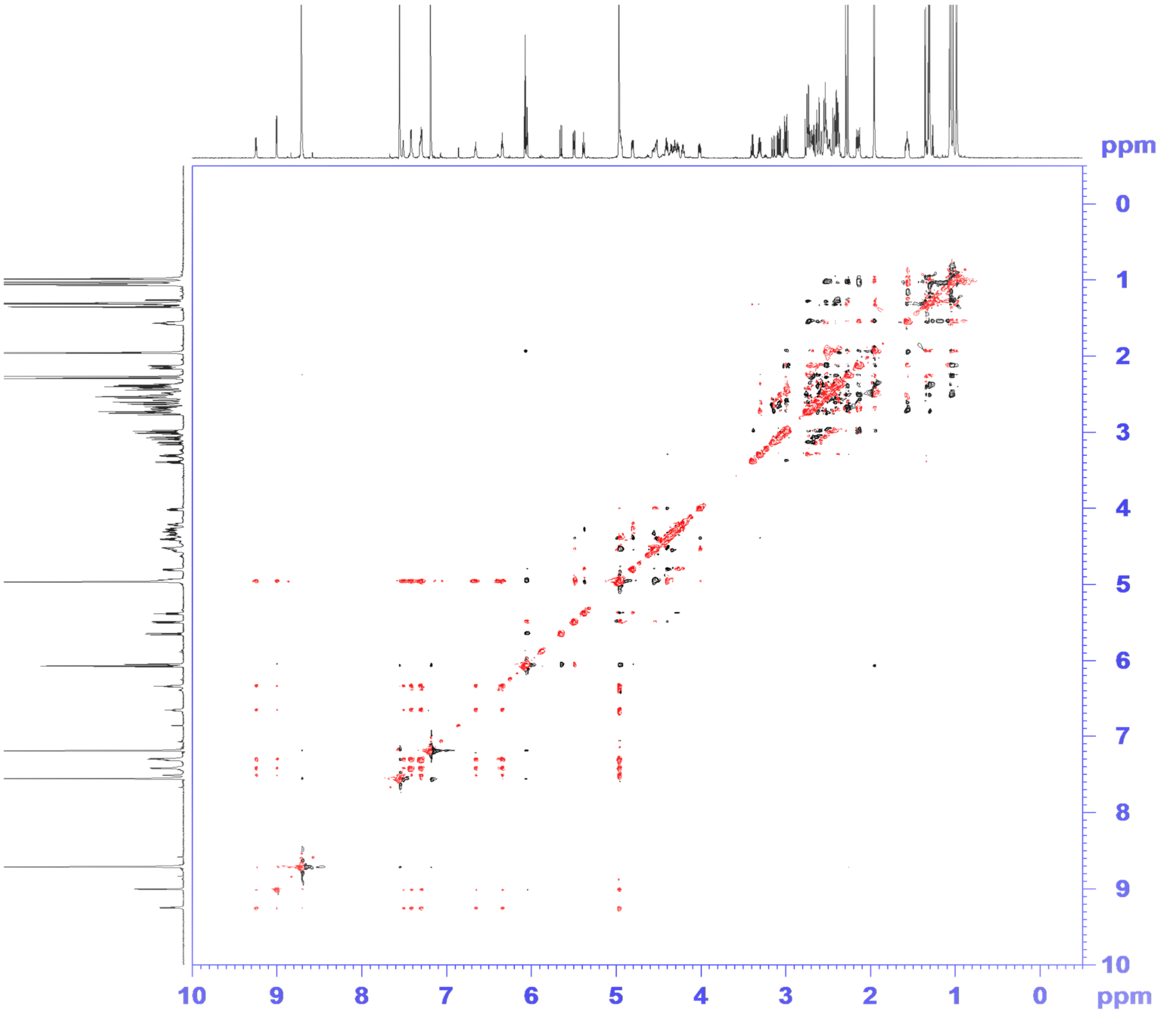

Figure S9. 2D NMR spectrum (^{1}H-^{1}H NOESY, 700 MHz, pyridine-d_5) of the compound (**1**).

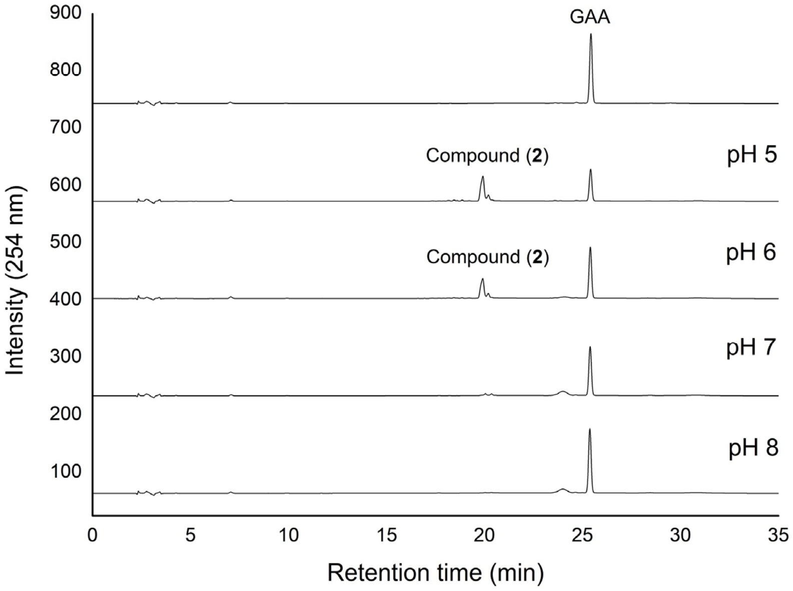

(**a**)

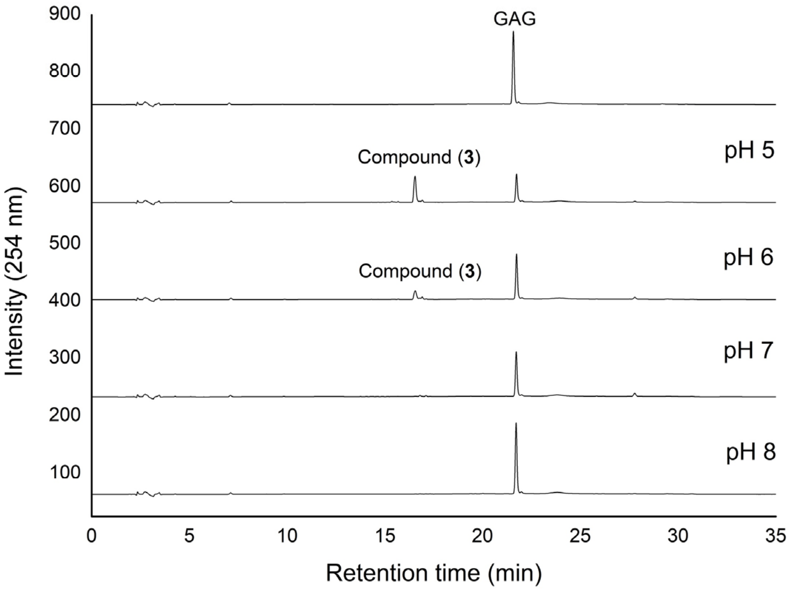

(**b**)

Figure S10. HPLC results of the biotransformation products of GAA (a) and GAG) (b) using *Dg*AS enzyme at different pH levels. The detailed reaction conditions and the HPLC procedure are the same as in Figure 2 and described in the Materials and Methods section.

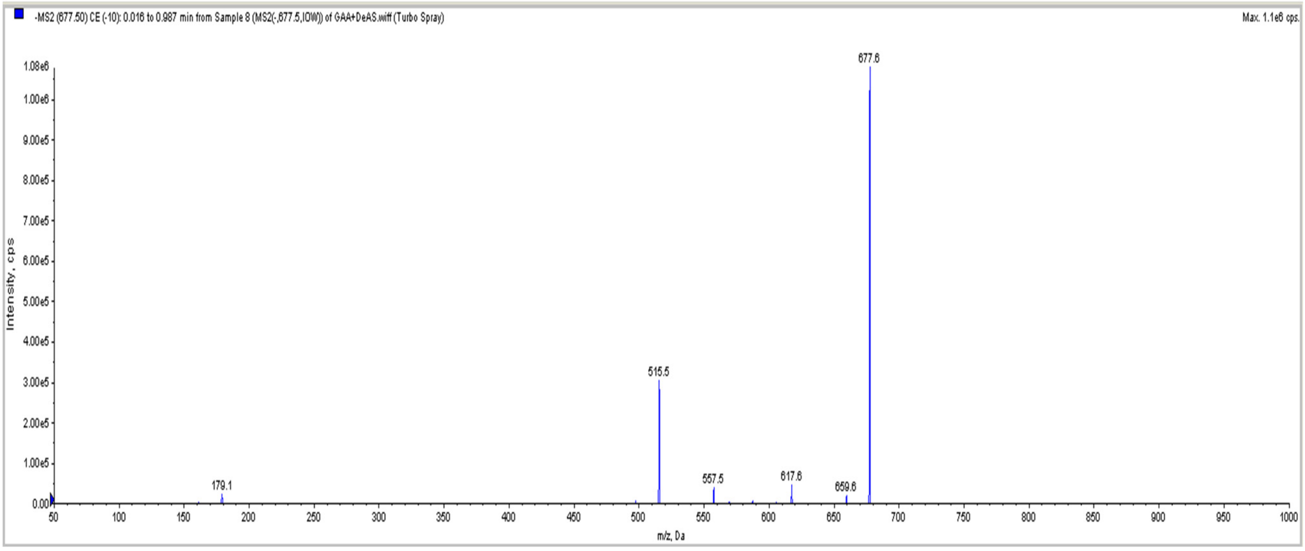

Figure S11. The mass-mass analysis of compound (**2**) at the negative mode. A significant signal at m/z 677.6 showed the corresponding m/z signal of molecular weight 678 of GAA-glucoside (516+180-18) at the negative mode.

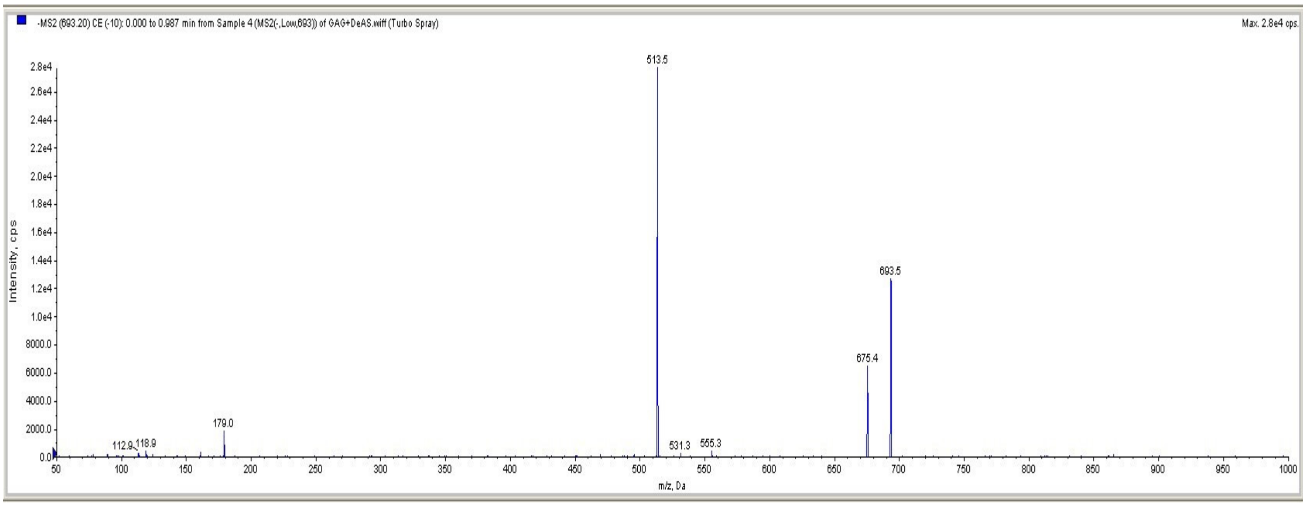

Figure S12. The mass-mass analysis of compound (**3**) at the negative mode. A significant signal at m/z 693.5 showed the corresponding m/z signal of molecular weight 694 of GAG-glucoside (532+180-18) at the negative mode.

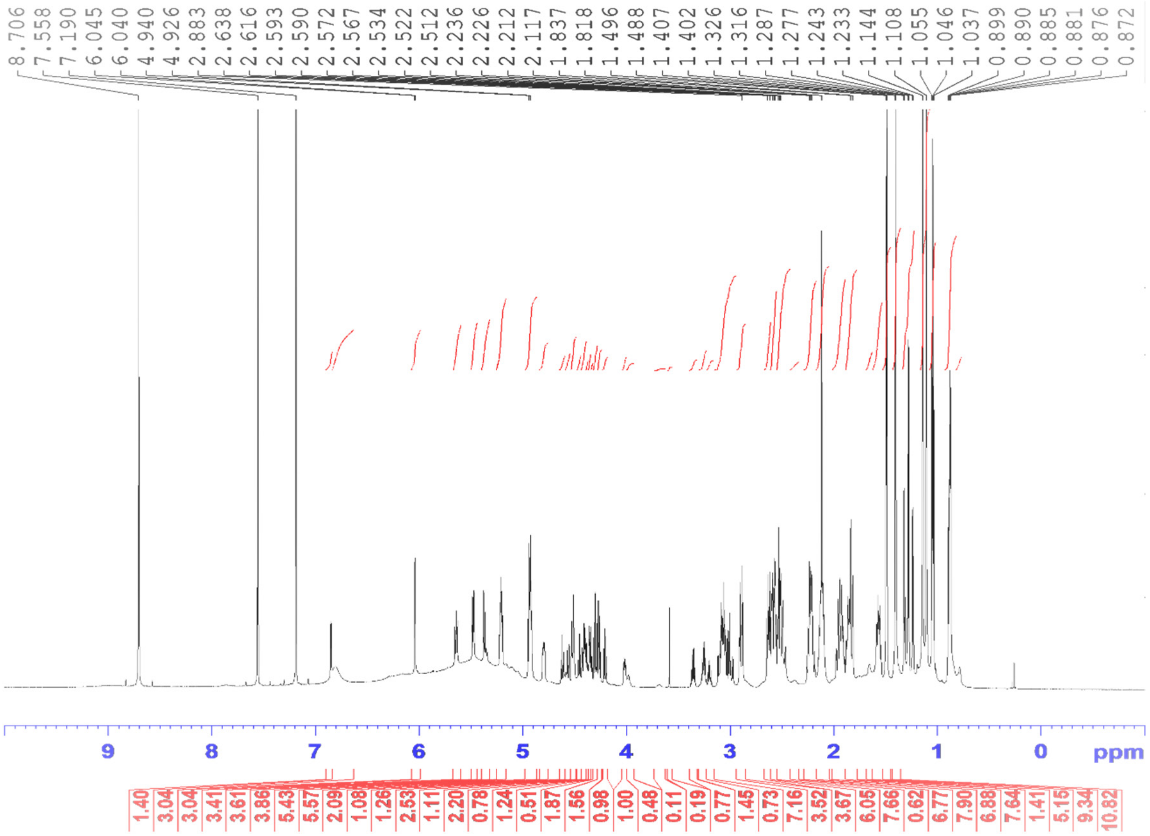

Figure S13. 1D NMR spectrum (^{1}H-NMR, 700 MHz, pyridine-d_5) of the compound (**2**).

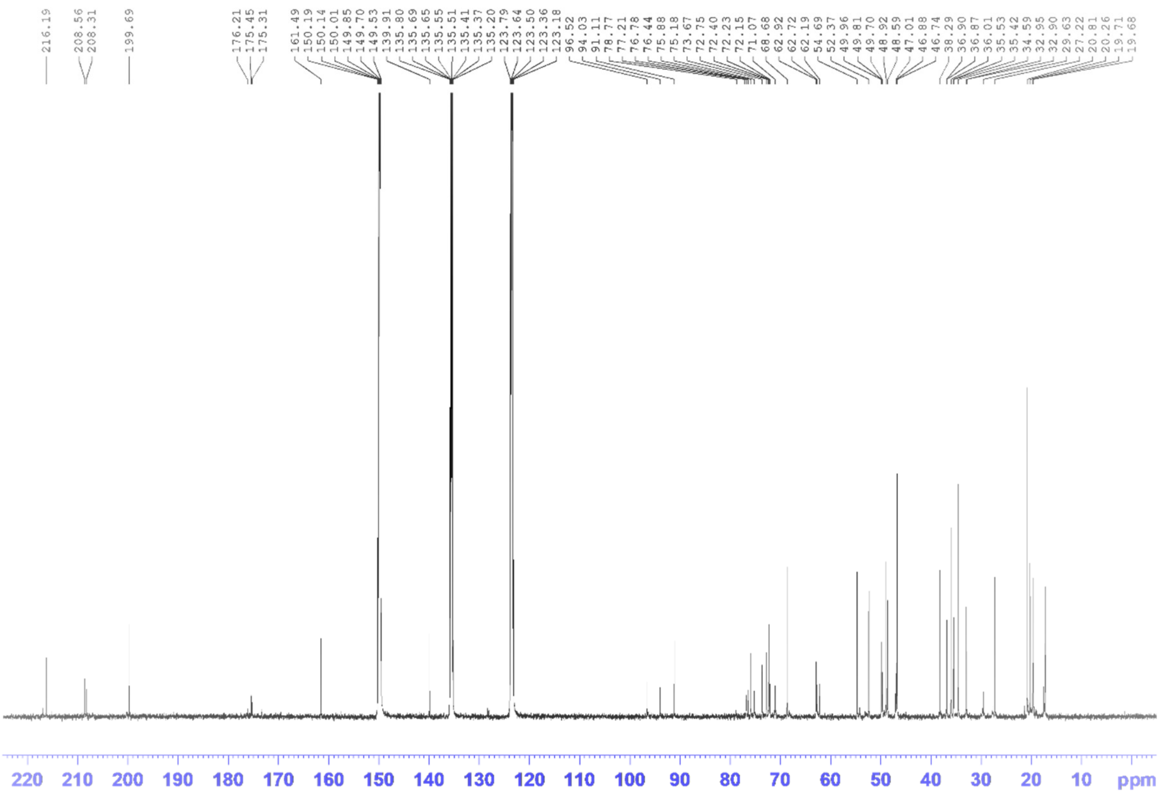

Figure S14. 1D NMR spectrum (^{13}C-NMR, 175 MHz, pyridine-d_5) of the compound (**2**).

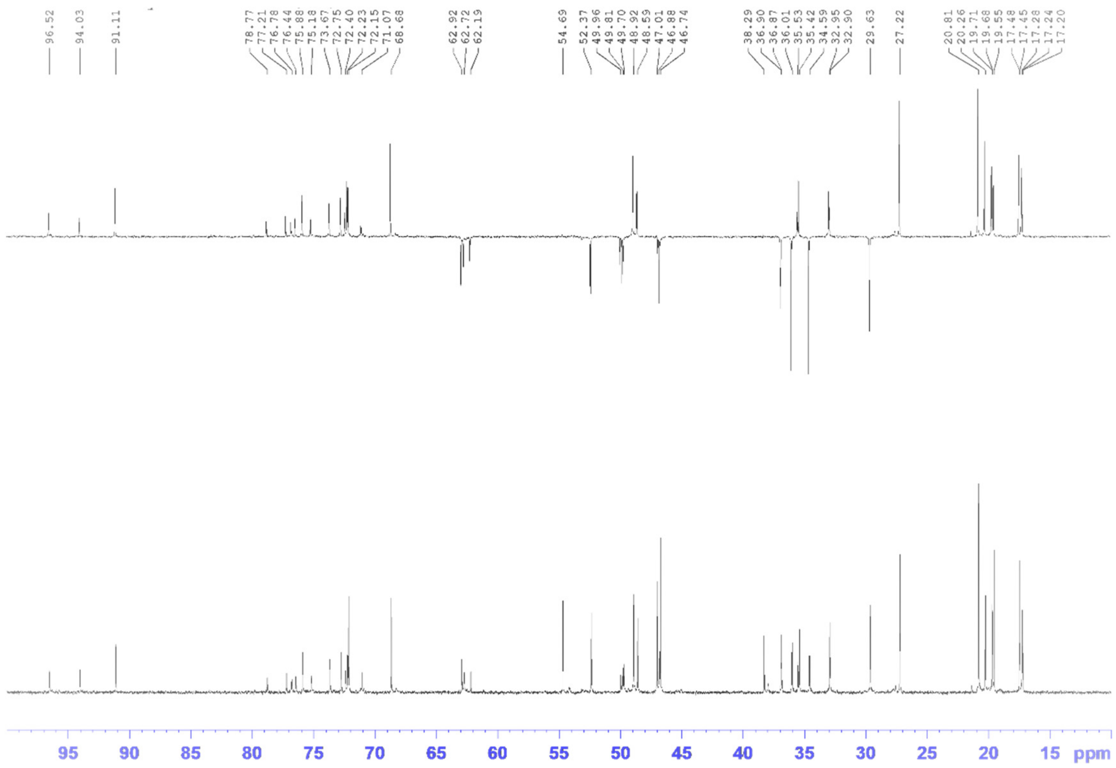

Figure S15. 1D NMR spectrum (DEPT-135, 175 MHz, pyridine-d_5) of the compound (**2**).

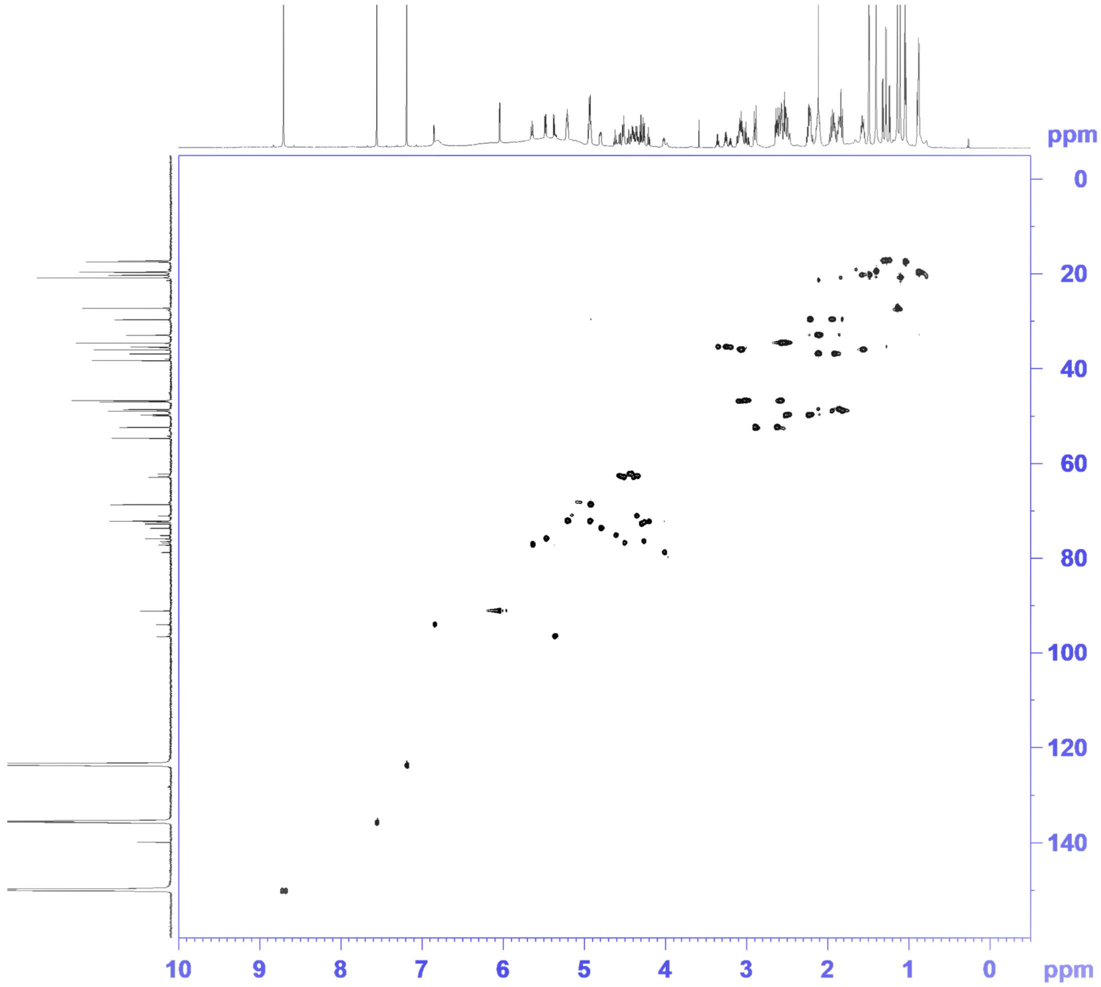

Figure S16. 2D NMR spectrum (^{1}H-^{13}C HSQC, 700 MHz, pyridine-d_5) of the compound (**2**).

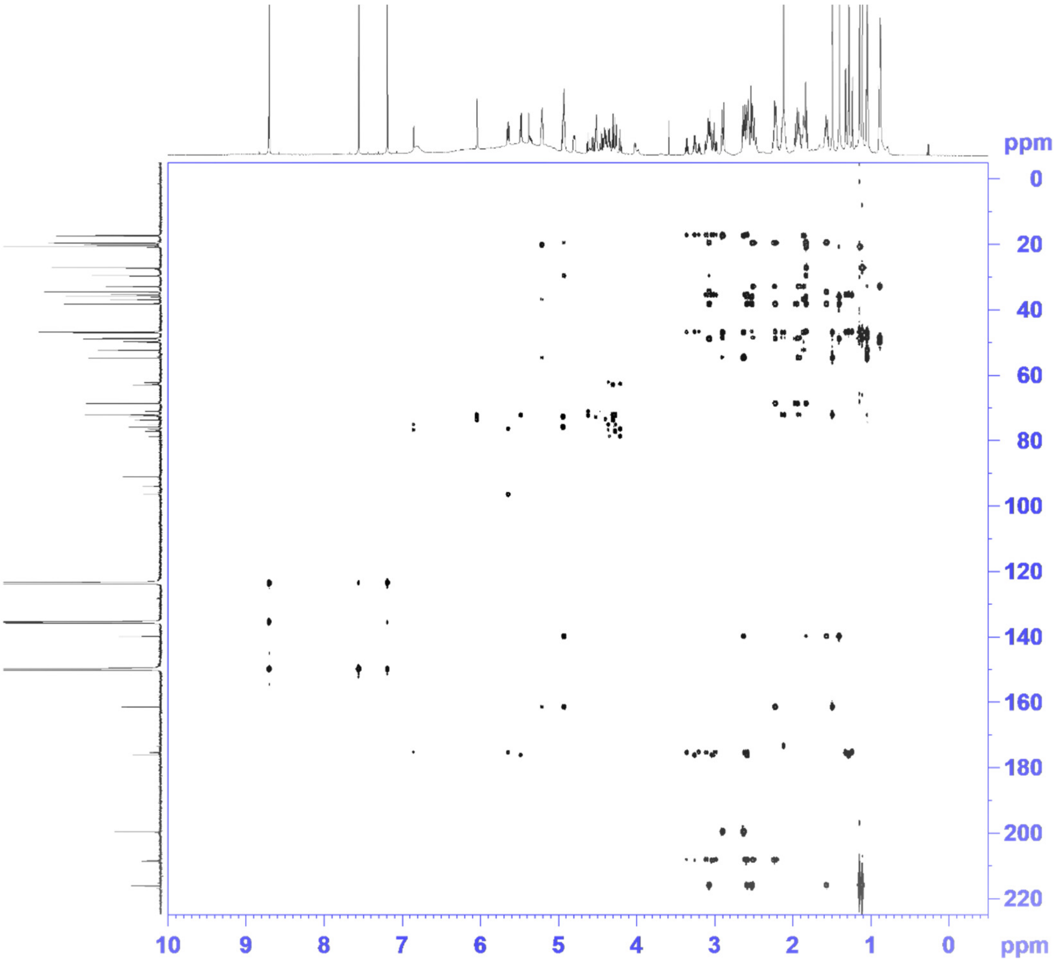

Figure S17. 2D NMR spectrum (^{1}H-^{13}C HMBC, 700 MHz, pyridine-d_5) of the compound (**2**).

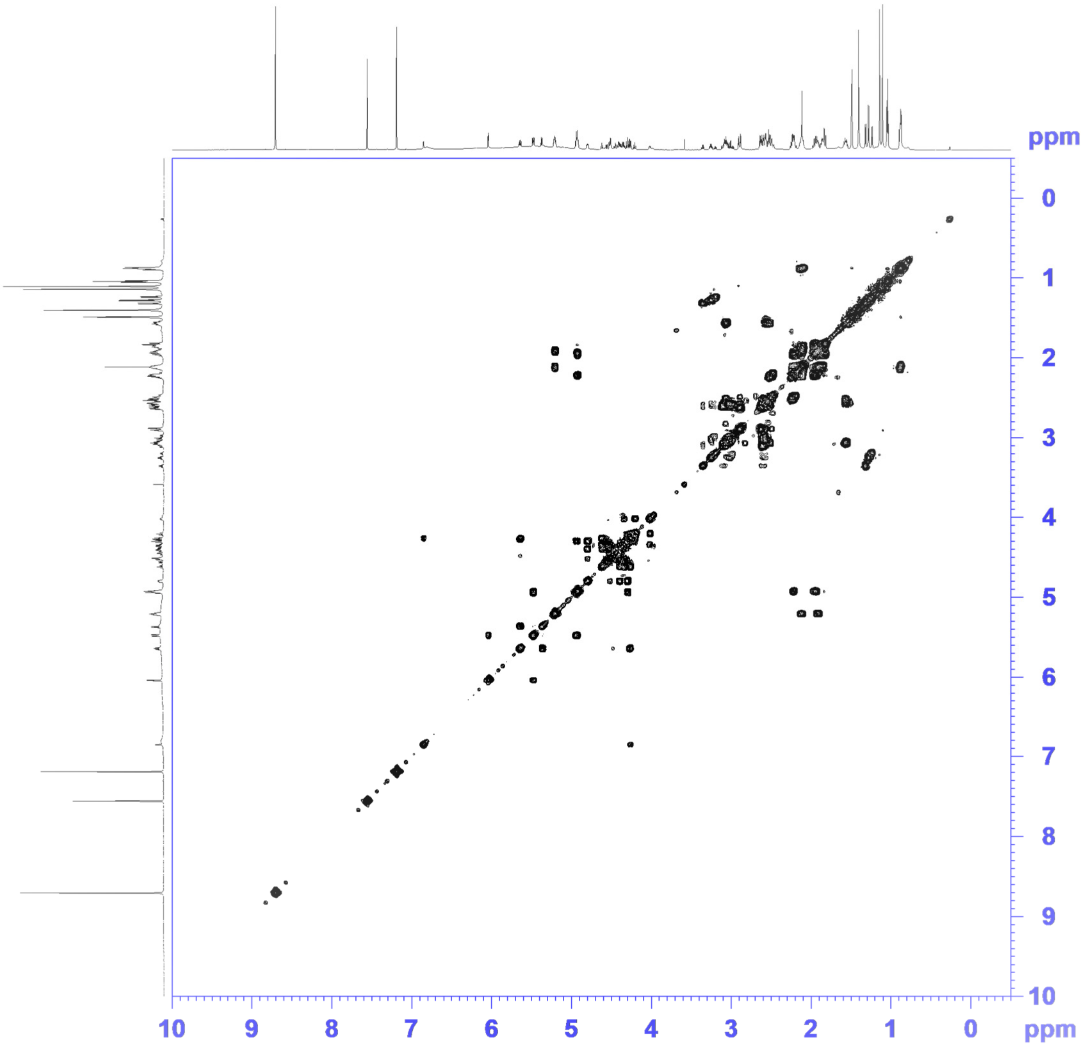

Figure S18. 2D NMR spectrum (^1H-^1H COSY, 700 MHz, pyridine-d_5) of the compound (**2**).

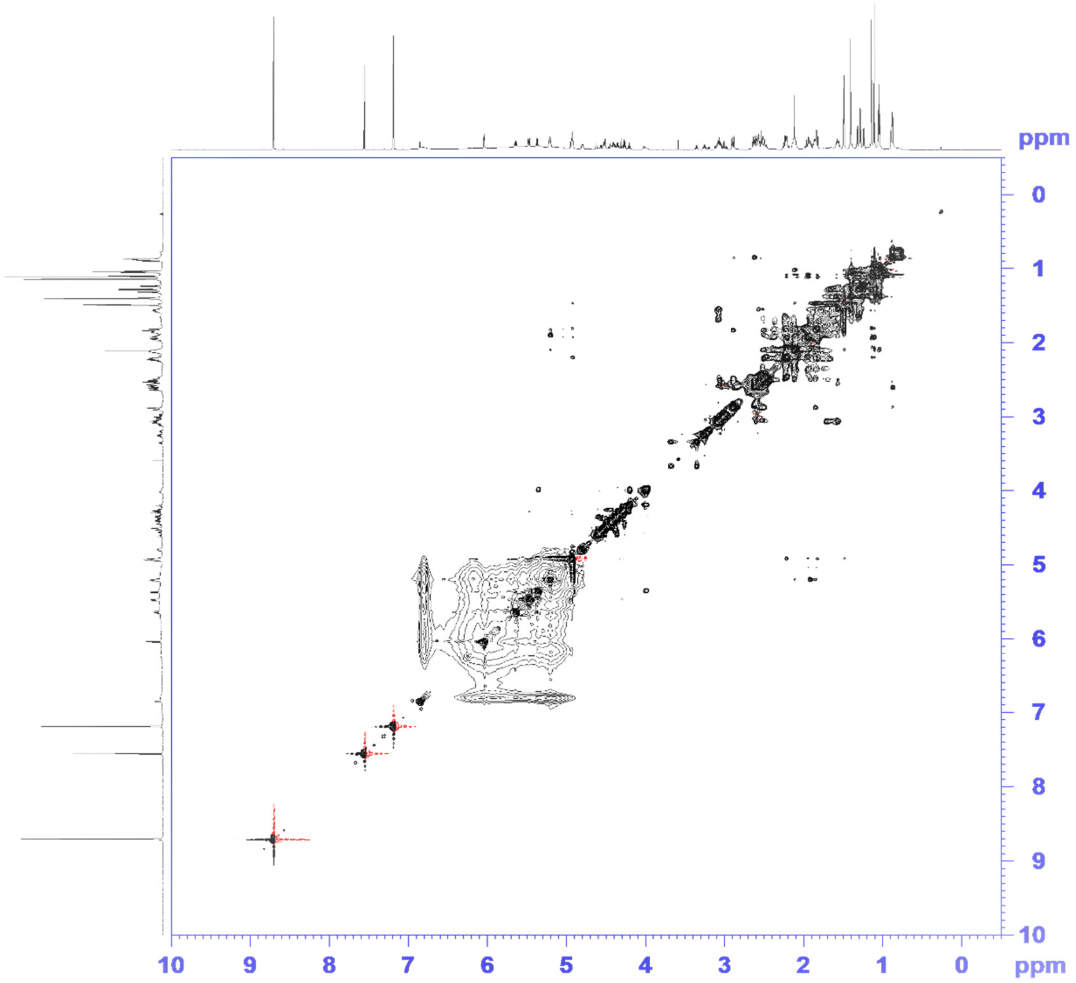

Figure S19. 2D NMR spectrum (^{1}H-^{1}H NOESY, 700 MHz, pyridine-d_5) of the compound (**2**).

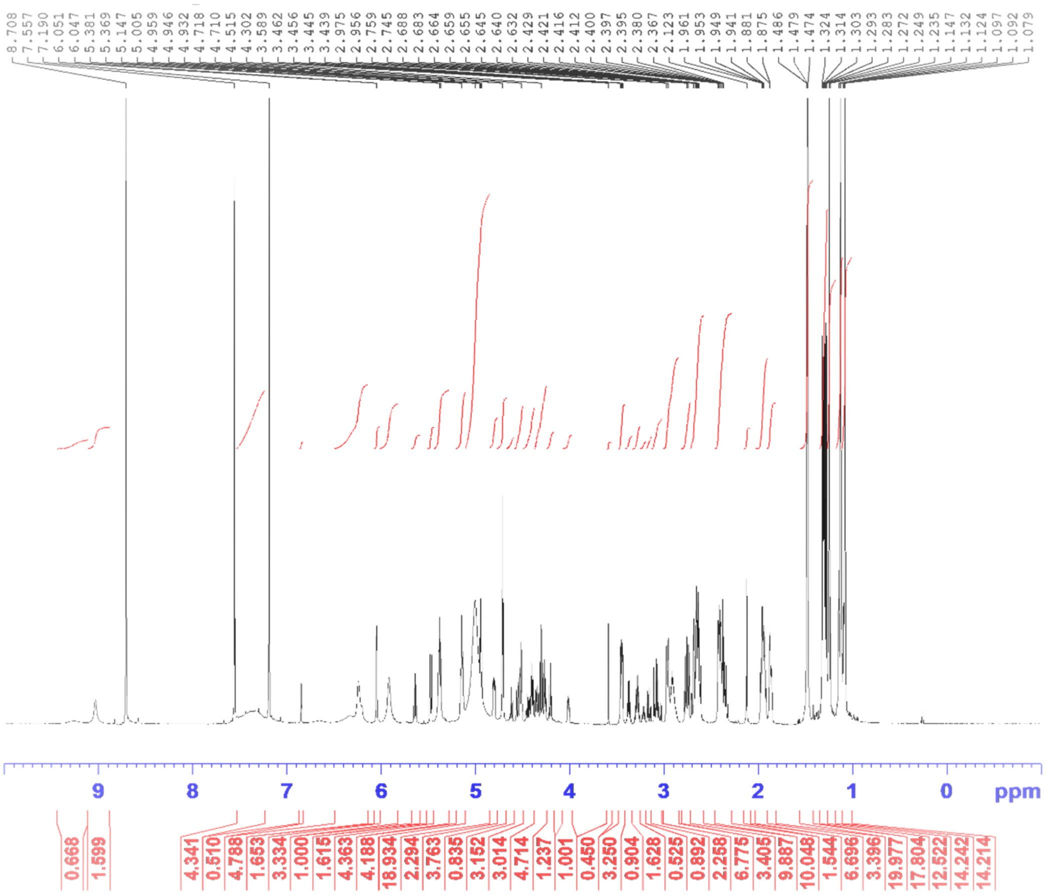

Figure S20. 1D NMR spectrum (^{1}H-NMR, 700 MHz, pyridine-d_5) of the compound (**3**).

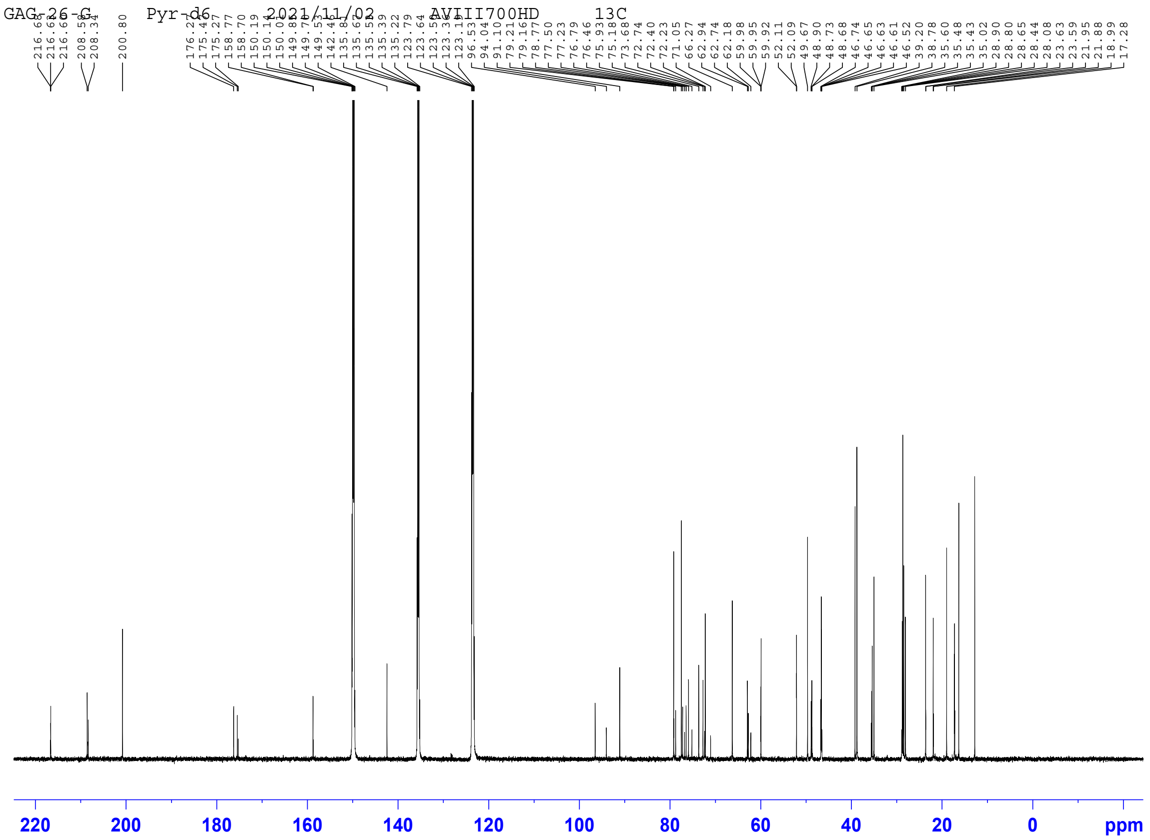

Figure S21. 1D NMR spectrum (^{13}C-NMR, 175 MHz, pyridine-d_5) of the compound (**3**).

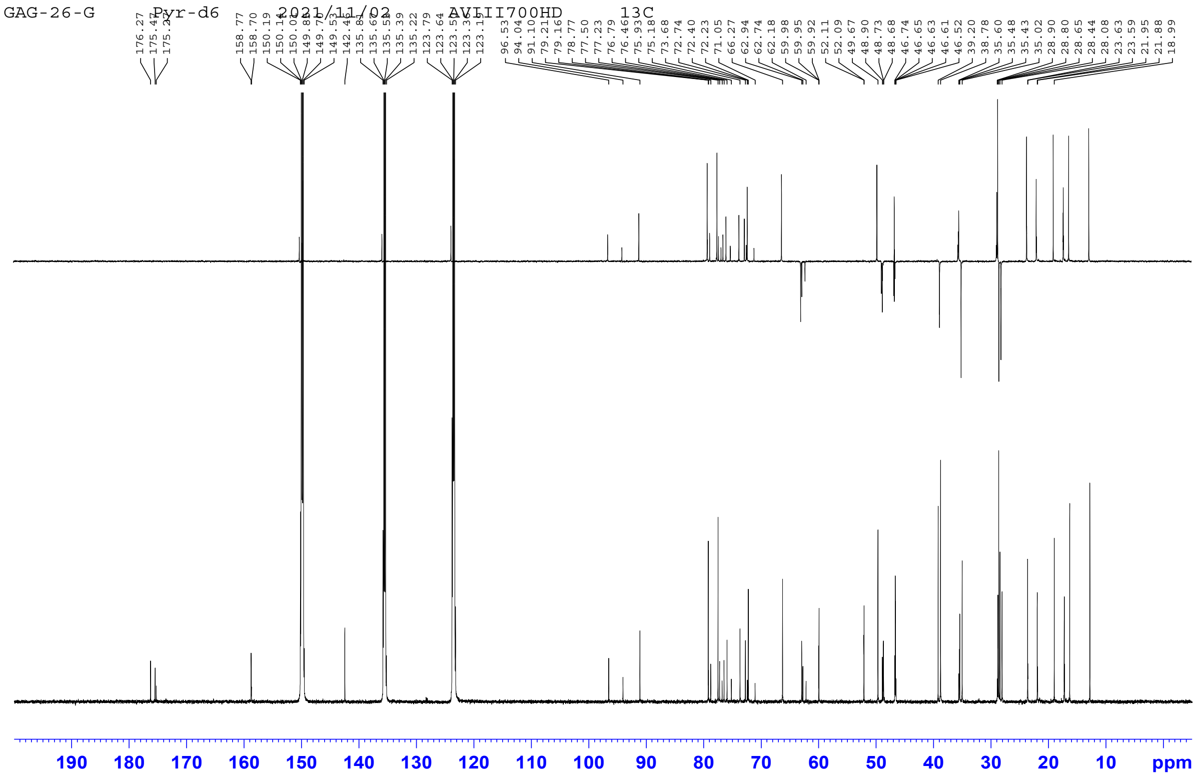

Figure S22. 1D NMR spectrum (DEPT-135, 175 MHz, pyridine-d_5) of the compound (**3**).

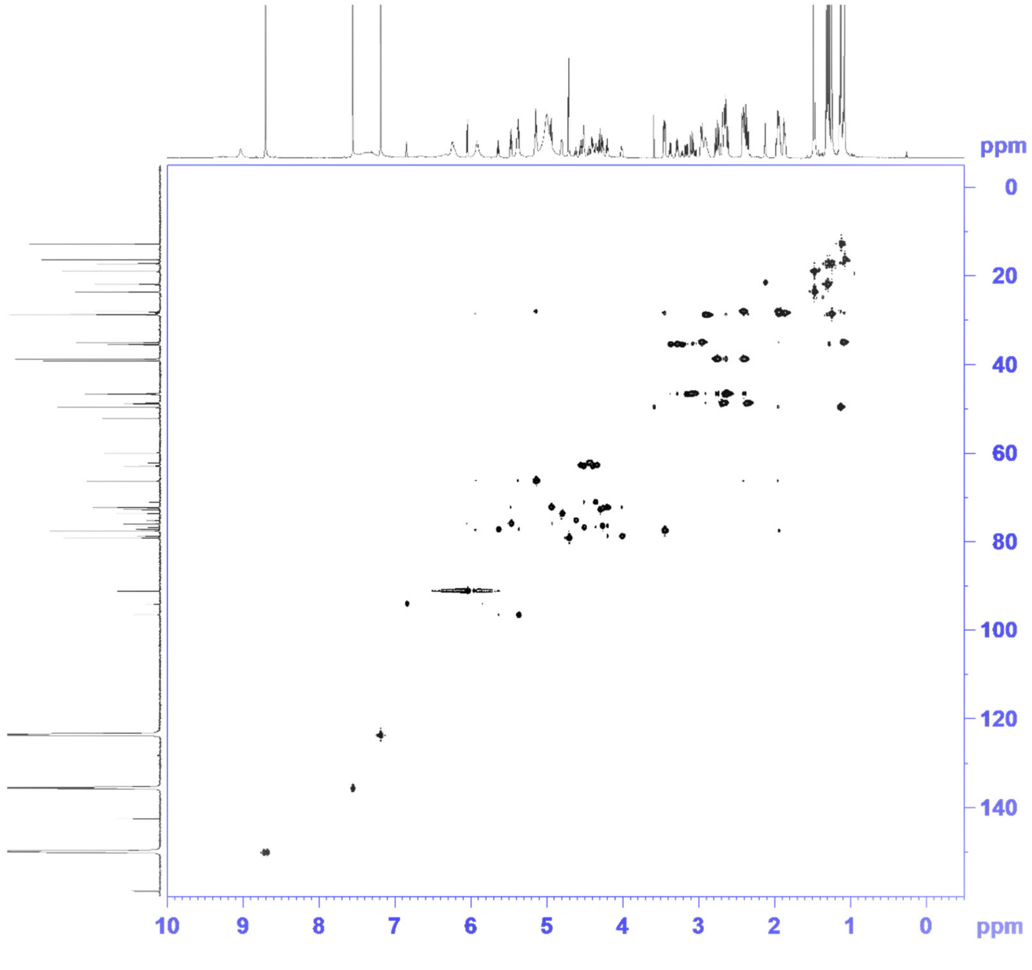

Figure S23. 2D NMR spectrum (^{1}H-^{13}C HSQC, 700 MHz, pyridine-d_5) of the compound (**3**).

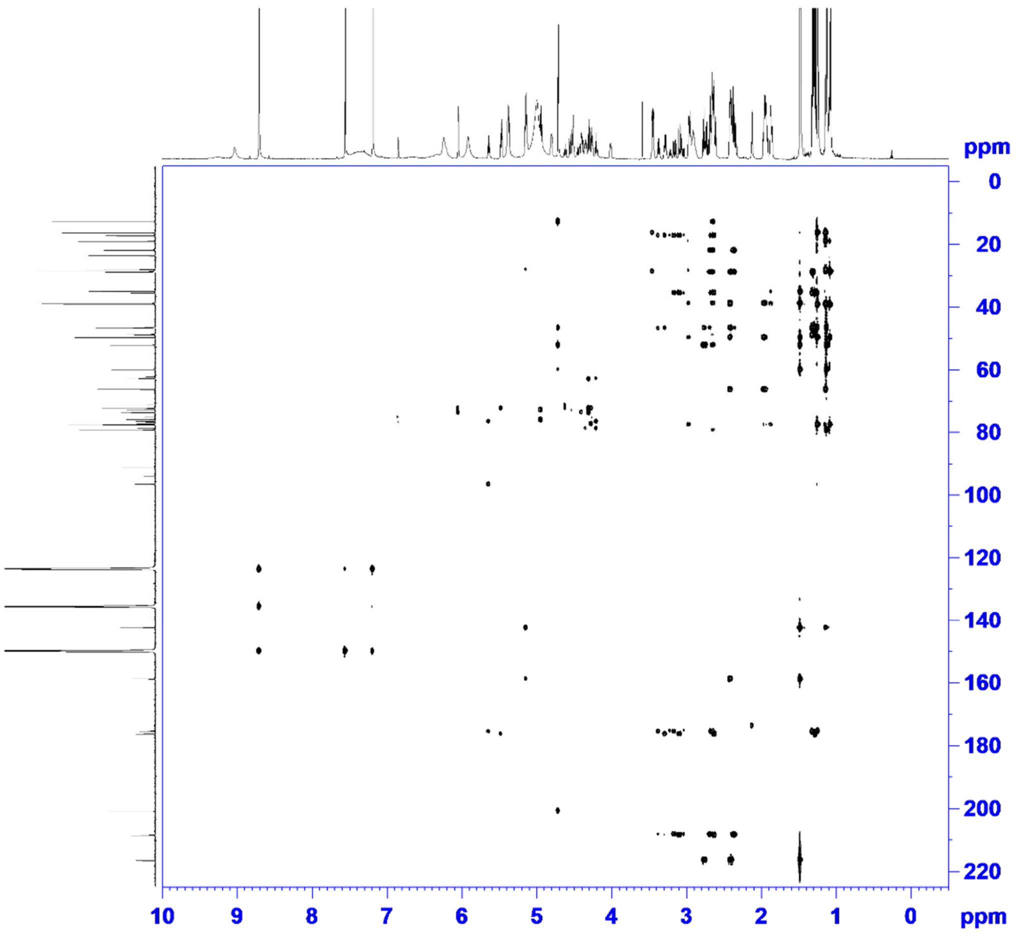

Figure S24. 2D NMR spectrum (^{1}H-^{13}C HMBC, 700 MHz, pyridine-d_5) of the compound (**3**).

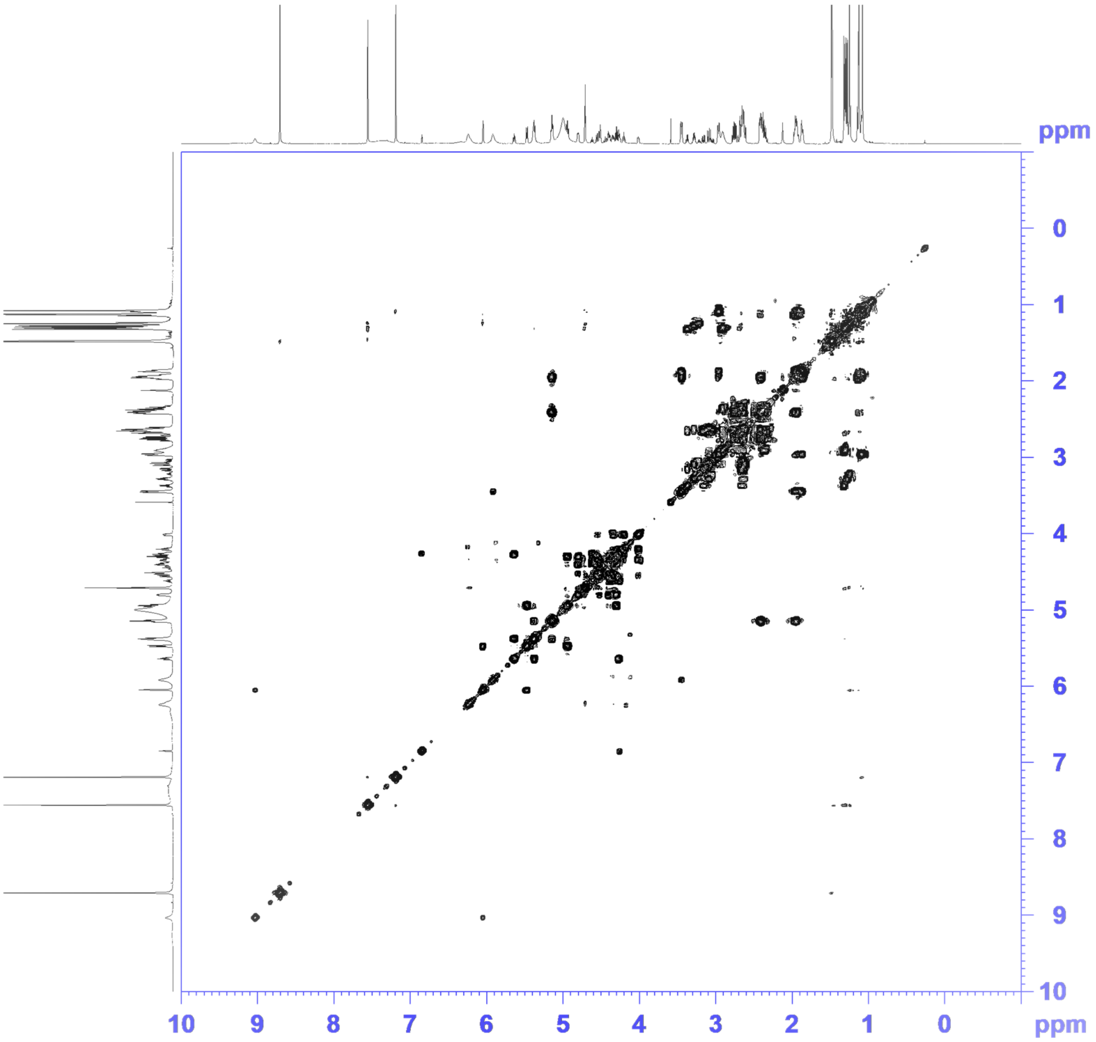

Figure S25. 2D NMR spectrum (^1H-^1H COSY, 700 MHz, pyridine-d_5) of the compound (**3**).

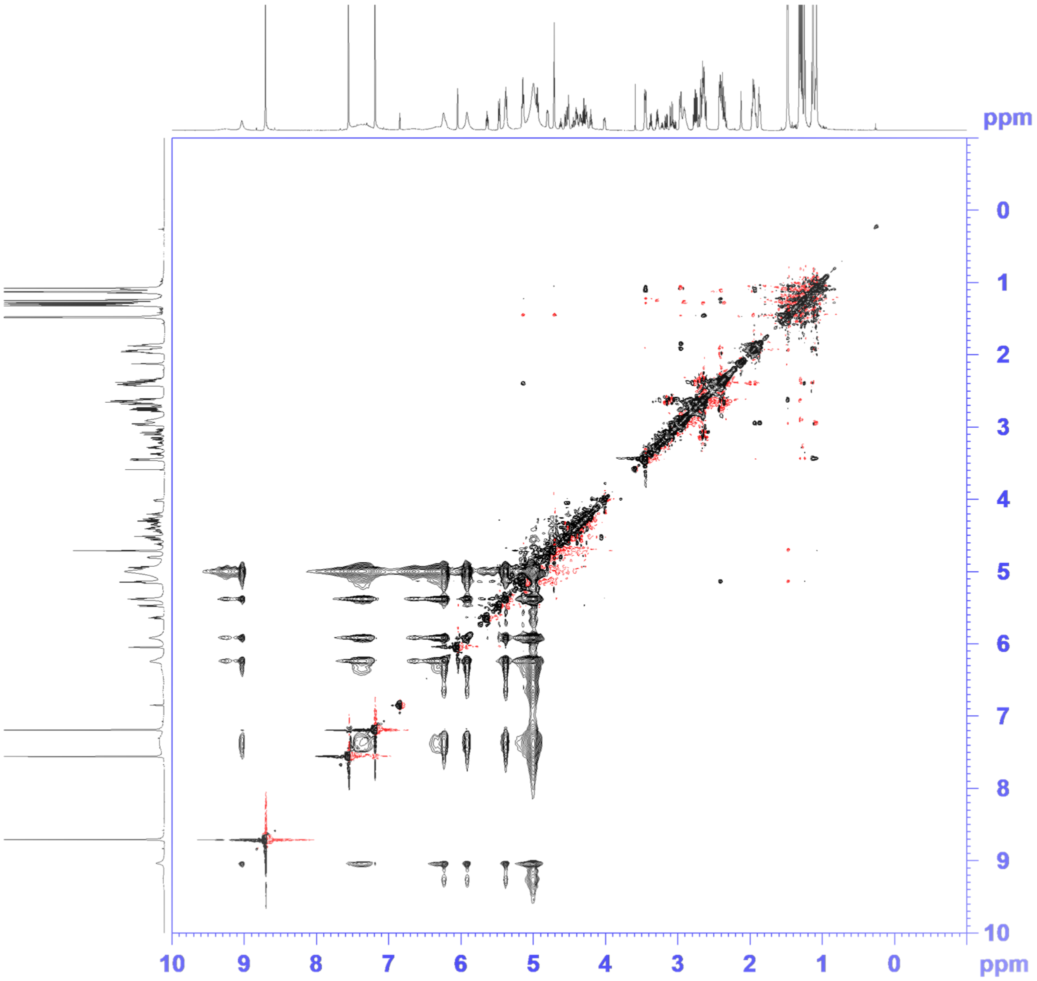

Figure S26. 2D NMR spectrum (^1H-^1H NOESY, 700 MHz, pyridine-d_5) of the compound (**3**).
